# Therapeutic Lymph Node Dissection in Melanoma: Different Prognosis for Different Macrometastasis Sites?

**DOI:** 10.1245/s10434-012-2401-8

**Published:** 2012-05-17

**Authors:** K. P. Wevers, E. Bastiaannet, H. P. A. M. Poos, R. J. van Ginkel, J. T. Plukker, H. J. Hoekstra

**Affiliations:** Division of Surgical Oncology, University Medical Center Groningen, University of Groningen, Groningen, The Netherlands

## Abstract

**Background:**

The prognostic significance of primary tumor location, especially the poor prognosis for melanomas in the scalp and neck region, is well established. However, the prognosis for different sites of nodal macrometastasis has never been studied. This study investigated the prognostic value of the location of macrometastasis in terms of recurrence and survival rates after therapeutic lymph node dissection (TLND).

**Methods:**

All consecutive FDG-PET-staged melanoma patients with palpable and cytologically proven lymph node metastases operated at our clinic between 2003 and 2011 were included. Disease-free survival and disease-specific survival (DSS) were compared for nodal metastases in the groin, axilla, and neck regions by multivariable analysis.

**Results:**

A total of 149 patients underwent TLND; there were 70 groin (47 %), 57 axillary (38 %), and 22 neck (15 %) dissections. During a median follow-up of 18 (range 1–98) months, 102 patients (68 %) developed recurrent disease. Distant recurrence was the first sign of progressive disease in 78, 76, and 55 % of the groin, axilla, and neck groups, respectively (*p* = 0.26). Low involved/total lymph nodes (L/N) ratio (*p* < 0.001) and absence of extranodal growth pattern (*p* = 0.05) were independent predictors of a longer disease-free survival. For DSS, neck site of nodal metastasis (*p* = 0.02) and low L/N ratio (*p* < 0.001) were independent predictors of long survival. The estimated 5-year DSS for the groin, axilla, and neck sites was 28, 34, and 66 %, respectively.

**Conclusions:**

There seems significantly longer DSS after TLND for nodal macrometastases in the neck compared to axillary and groin sites, although larger series should confirm this finding.

The incidence of melanoma continues to increase in the Western world. In the Netherlands, the incidence doubled over the past two decades, to 26.3 per 100,000 in 2009 from 11.3 per 100,000 in 1989.^1^,^2^ Most patients present initially with stage I or II melanoma.^3^ Unfortunately, despite defined surgical treatment of the primary melanoma with excision margins of 1 or 2 cm, approximately 16–28 % of patients develop recurrent disease. These recurrences occur locally or in transit in 20–28 %, distant in 15–50 %, but most frequently in regional lymph nodes (26–60 %).^4^

When nodal recurrence is detectable clinically (stage IIIB–C), patients may benefit from therapeutic lymph node dissection (TLND) with or without adjuvant radiation treatment in terms of regional tumor control and survival, resulting in a 5-year survival rate of 29–52 %.^3^,^5^–^9^ Major predictors of an unfavorable prognosis are greater Breslow thickness, the presence of ulceration, and a high mitotic rate. Clark level, location of the primary melanoma, age, and sex are less important predictors.^3^,^10^ The prognostic significance of primary melanoma characteristics cannot be identified for patients with nodal metastasis undergoing TLND.^5^ For this group of patients, a recent study showed that a preoperatively elevated S-100B tumor marker had a negative prognostic value.^11^

The prognostic significance of primary tumor location, especially worse prognosis for melanomas in the scalp and neck region, is well established.^12^,^13^ However, the prognostic value of the anatomic location of nodal recurrence in stage IIIB–C melanoma has not previously been investigated. Patients with nodal metastasis are at high risk for distant metastasis. Therefore, patients with stage III melanoma and palpable lymph node metastases are staged by whole body FDG-PET and spiral CT at our center since the last decade, avoiding unnecessary surgery in the presence of systemic disease in 15.5 % of these patients.^14^

The aim of the present study was to analyze the site of recurrence, the disease-free survival (DFS), and the disease-specific survival (DSS) according to the anatomic location of lymph node metastasis (groin, axilla, and neck) in optimally staged patients with stage IIIB–C melanoma.

## Patients and Methods

All consecutive melanoma patients with palpable and cytologically proven lymph node metastases diagnosed at the Division of Surgical Oncology of the University Medical Center Groningen (UMCG), the Netherlands, between 2003 and 2011 underwent staging with whole-body FDG-PET and spiral CT. All patients were informed about their stage of disease, type of regional nodal dissection, and potential perioperative complications, according to the UMCG standards. Those with distant metastases or with more than one affected lymph node basin were excluded from this study. A total of 149 stage IIIB–C melanoma patients underwent a TLND. In this group, only 7 patients had been staged previously with sentinel lymph node biopsy, which was negative in 6 cases. The single patient with a positive sentinel lymph node biopsy refused a proposed completion lymph node dissection at the time and experienced disease recurrence later in the affected regional lymph node basin.

All therapeutic dissections were performed by experienced surgical oncologists. A level I–III axillary dissection was performed with resection of the minor pectoral muscle. Groin dissection comprised superficial (inguinal) and deep (iliac and obturator) lymph node dissection with sartorius muscle transposition.^15^ Neck dissection included radical removal of lymph nodes in levels I–III, I–V, and II–V, including the posterior compartment depending on indication. A subtotal dissection of the parotid gland was performed depending on the localization of the lymph node metastasis and the primary site.

Patients with positive lymph nodes larger than 3 cm, three or more positive lymph nodes, and/or extranodal growth pattern received adjuvant radiotherapy (45–60 Gy).^16^,^17^

All patients with recurrence after TLND were discussed in a multidisciplinary melanoma conference and received tailored treatment (surgery, radiotherapy, and/or systemic treatment) according to the current standard or experimental treatment protocols.

Characteristics of the patient (sex and age), primary melanoma (Breslow thickness, Clark level, ulceration, mitotic rate, and primary disease site), and lymph node metastasis (interval to metastasis, extranodal growth pattern, total number of nodes, number of involved nodes, involved/total lymph nodes (L/N) ratio, and size of the largest nodal metastasis) were recorded and analyzed for differences between the groin, axillary, and neck groups. Fisher’s exact test or the chi-square test for categorical variables and the Kruskal–Wallis test for continuous variables were used to analyze the differences by using a significance level of 5 %. DFS and DSS were calculated from the date of the TLND. Univariate and multivariable Cox proportional hazards analysis, as well as Kaplan-Meier curves, were used to assess DFS and DSS for different nodal metastasis locations, with an event defined as any recurrence for DFS and death due to melanoma for DSS. All factors significant at a 10 % significance level in univariate analysis were included in a multivariable model along with sex, age, Breslow thickness, and ulceration. Quantitative characteristics were entered as continuous variables in univariate and multivariable analysis on DFS and DSS. Because of its prognostic significance, we used the L/N ratio rather than the number of involved nodes for multivariable analysis.^18^–^20^ A backward stepwise method was then used to identify independent predictors for DFS and DSS at the 5 % significance level.

## Results

A total of 149 patients underwent TLND. There were 70 groin dissections (47 %), 57 axillary dissections (38 %), and 22 neck dissections (15 %). The median age was 58 (range 16–93) years, and 64 patients (43 %) were female.

Significant differences in characteristics between the three lymph node basin groups were found for sex (*p* = 0.001, with more males in the axilla and neck groups), Clark level (*p* = 0.05, lower in neck group), total number of collected nodes (*p* = 0.04, higher in neck group), and size of largest lymph node metastasis at pathologic examination (*p* < 0.001, with smaller metastases in the neck group) (Table 1).

**Table 1:** Patient characteristics according to location of lymph node metastasis

Characteristic	No. of patients (%)			
	Groin	Axilla	Neck	*p*
Sex				
Female	41 (59)	19 (33)	4 (18)	**0.001**
Male	29 (41)	38 (67)	18 (82)	
Age (year)				
Median (range)	58 (29–87)	53 (25–93)	59 (16–82)	0.26
<50	17 (24)	24 (42)	7 (32)	
50–64	32 (46)	19 (33)	5 (23)	
65+	21 (30)	14 (25)	10 (45)	
Breslow thickness (mm)				
Median (range)	2.1 (0.1–16)	1.8 (0.4–8)	2.5 (0.5–14)	0.51
T1 (<1.00)	6 (9)	9 (16)	3 (13)	
T2 (1.00–2.00)	24 (34)	18 (32)	5 (23)	
T3 (2.00–4.00)	26 (37)	15 (26)	5 (23)	
T4 (>4.00)	9 (13)	7 (12)	5 (23)	
Unknown	5 (7)	8 (14)	4 (18)	
Clark level				
II/III	10 (17)	11 (26)	6 (32)	**0.05**
IV/V	45 (75)	26 (62)	7 (36)	
Unknown	5 (8)	5 (12)	6 (32)	
Unknown primary melanoma				
No	67 (96)	52 (91)	19 (86)	0.27
Yes	3 (4)	5 (9)	3 (14)	
Ulceration				
Absent	42 (60)	30 (52)	14 (64)	0.16
Present	24 (34)	18 (32)	2 (9)	
Unknown	4 (6)	9 (16)	6 (27)	
Mitotic rate per mm^2^				
Median (range)	5 (0–18)	4 (0–21)	4 (1–35)	0.89
<5	28 (40)	28 (49)	8 (36)	
≥5	29 (41)	19 (33)	6 (28)	
Unknown	13 (19)	10 (18)	8 (36)	
Interval between primary melanoma and nodal metastasis (year)^a^				
Median (range)	2.1 (0–17)	1.9 (0–15)	1.2 (0–19)	0.65
≤2 years	32 (48)	28 (54)	11 (58)	
>2 years	35 (52)	24 (46)	8 (42)	
Extranodal growth pattern				
No	36 (51)	39 (68)	12 (54)	0.14
Yes	34 (49)	18 (32)	10 (46)	
Total no. of nodes				
Median (range)	15 (2–38)	16 (6–43)	24 (3–70)	**0.04**
No. of involved nodes^b^				
Median (range)	3 (1–23)	2 (1–25)	2 (1–10)	0.27
N1 (1)	21 (30)	25 (44)	8 (36)	
N2 (2–3)	22 (31)	15 (26)	7 (32)	
N3 (4+)	27 (39)	7 (32)	7 (32)	
Ratio of involved/total nodes (%)				
Median (range)	15 (3–100)	15 (2–100)	10 (1–67)	0.12
≤10	24 (34)	24 (42)	11 (50)	
10–25	19 (27)	18 (32)	8 (36)	
>25	27 (39)	15 (26)	3 (7)	
Size of nodal metastasis (cm)				
Median (range)	2.8 (0.1–7.0)	5.0 (1.5–9.0)	2.2 (0.3–6.0)	**<0.001**
<3.0	35 (50)	14 (25)	16 (73)	
≥3.0	33 (47)	41 (72)	5 (23)	
Unknown	2 (3)	2 (3)	1 (4)	
AJCC stage^b^				
IIIB	26 (37)	26 (46)	14 (64)	0.09
IIIC	44 (63)	31 (54)	8 (36)	
Duration of follow-up (months)				
Median (range)	19 (1–93)	16 (1–98)	43 (3–94)	**0.05**

*p*-values below 0.05 in bold

^a^Unknown primary melanoma not included in calculation of interval

^b^According to the 7th melanoma classification of the American Joint Committee on Cancer

### Site of Recurrence

One hundred two patients (68 %) developed recurrent disease during follow-up. As shown in Table 2, a large proportion of patients in the groin and axilla groups had recurrent disease and presented with distant metastases as the first sign of progressive disease (78 and 76 %). In the neck group, only 55 % of patients presented with a distant metastasis as the first site of recurrence (*p* = 0.26).

**Table 2:** Site of first recurrence after therapeutic lymph node dissection according to location of lymph node metastasis

Site of recurrence^a^	Local	Locoregional	Distant	*p*
Groin	2 (4)	10 (18)	42 (78)	0.26
Axilla	4 (11)	5 (13)	28 (76)	
Neck	1 (9)	4 (36)	6 (55)	

^a^Patients presenting with both local or locoregional and distant recurrences were classified as distant

### Recurrence and Survival Rates

**Fig. 1:**
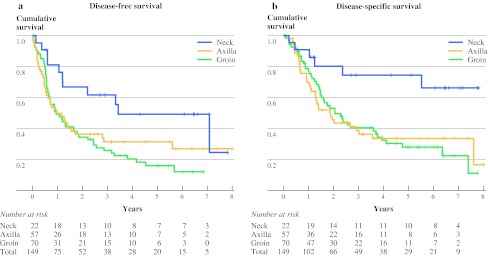
Kaplan–Meier curves for disease-free survival (**a**) and disease-specific survival (**b**) according to location of lymph node metastasis

The follow-up for the entire group was 18 (range 1–98) months with an estimated 5-year DFS of 27 % (95 % confidence interval 19–34) and an estimated 5-year DSS of 37 % (95 % confidence interval 28–45). The estimated 5-year DFS for the groin, axilla, and neck groups was 12, 27, and 49 %, respectively (Fig. 1a). Variables associated with DFS in univariate analysis were presence of ulceration, the location of nodal metastasis, extranodal growth pattern, L/N ratio, and the size of the largest nodal metastasis. Neck location of the metastasis showed a significantly longer DFS in univariate analysis (Table 3). The multivariable model showed a lower L/N ratio (*p* < 0.001) and an absence of extranodal growth pattern (*p* = 0.05) to be independent predictors of longer DFS. The association of the location of lymph node metastasis with DFS was not statistically significant in the multivariable model (Table 4).

**Table 3:** Univariate Cox regression analysis of prognostic factors for DFS and DSS

Characteristic	DFS			DSS		
	HR	95 % CI	*p*	HR	95 % CI	*p*
Sex						
Female	1			1		
Male	1.07	0.72–1.59	0.73	1.28	0.83–1.98	0.26
Age (years)						
Continuous	0.99	0.98–1.00	0.15	1.00	0.98–1.01	0.70
<50	1			1		
50–64	0.93	0.58–1.49		1.07	0.64–1.80	
≥65	0.88	0.54–1.43		1.15	0.67–1.99	
Breslow thickness (mm)						
Continuous	0.98	0.89–1.07	0.60	0.98	0.89–1.09	0.74
T1 (<1.00)	1			1		
T2 (1.00–2.00)	0.79	0.40–1.56		1.10	0.50–2.42	
T3 (2.00–4.00)	1.09	0.56–2.09		1.17	0.53–2.57	
T4 (>4.00)	0.73	0.33–1.61		0.86	0.35–2.15	
Clark level						
II	1			1		
III	1.77	0.41–7.70	0.46	3.71	0.48–28.5	0.21
IV	2.55	0.63–10.44	0.20	4.86	0.67–35.2	0.12
V	2.29	0.46–11.34	0.32	3.60	0.40–32.4	0.25
Unknown primary melanoma						
No	1			1		
Yes	1,66	0.72–3.83	0.23	1.29	0.56–2.97	0.55
Location of primary melanoma						
Arm	1			1		
Leg	1.64	0.77–3.49	0.20	1.55	0.68–3.53	0.29
Trunk	1.42	0.65–3.08	0.37	1.59	0.69–3.67	0.28
Head/neck	0.54	0.19–1.57	0.26	0.56	0.17–1.73	0.31
Ulceration						
Present	1			1		
Absent	0.64	0.42–0.99	**0.05**	0.72	0.44–1.16	0.18
Mitotic rate per mm^2^						
Continuous	1.00	0.97–1.04	0.86	1.00	0.96–1.05	0.84
<5	1			1		
≥5	1.19	0.76–1.87		1.13	0.69–1.86	
Interval between primary melanoma and nodal metastasis (years)						
Continuous	0.95	0.90–1.01	0.11	0.90	0.88–1.01	0.09
Location metastasis						
Groin	1			1		
Axilla	0.84	0.55–1.28	0.42	0.99	0.63–1.56	0.97
Neck	0.42	0.22–0.81	**0.009**	0.34	0.15–0.77	**0.009**
Extranodal growth pattern						
No	1			1		
Yes	1.96	1.33–2.90	**0.001**	1.86	1.21–2.85	**0.004**
No. of involved nodes						
Continuous	1.06	1.03–1.10	**0.001**	1.07	1.03–1.12	**0.001**
N1 (1)	1			1		
N2 (2–3)	1.32	0.76–2.31		0.99	1.56–1.74	
N3 (4+)	2.42	1.42–4.11		2.33	1.40–3.88	
Ratio of involved/total nodes (%)						
Continuous	1.02	1.01–1.02	**<0.001**	1.01	1.01–1.02	**<0.001**
≤10	1			1		
10–25	1.27	0.73–2.22		1.25	0.71–2.18	
>25	2.30	1.37–3.88		2.69	1.60–4.52	
Size the lymph node metastasis (cm)						
Continuous	1.14	1.02–1.26	**0.02**	1.17	1.05–1.31	**0.004**
<3.0	1			1		
≥3.0	1.40	0.94–2.09		1.71	1.09–2.68	

*p*-values below 0.05 in bold

*DFS* disease-free survival, *DSS* disease-specific survival, *HR* hazard ratio, *CI* confidence interval

All variables with *p* < 0.10 were included in multivariable model along with sex, age, Breslow thickness, and ulceration

**Table 4:** Multivariable Cox regression analysis of prognostic value of nodal metastasis location for DFS and DSS

Site	DFS			DSS		
	5-year DFS, % (95 % CI)	Multivariable HR (95 % CI)^a^	*p*	5-year DSS, % (95 % CI)	Multivariable HR (95 % CI)^b^	*p*
Groin	12.1 (2.1–22.1)	1 (reference)		28.2 (16.0–40.3)	1 (reference)	
Axilla	27.1 (13.4–40.8)	0.94 (0.59–1.50)	0.78	33.6 (19.9–47.3)	0.98 (0.60–1.60)	0.93
Neck	49.2 (26.5–71.9)	0.48 (0.22–1.09)	0.08	66.3 (43.2–89.4)	0.27 (0.10–0.79)	**0.02**

*p*-values below 0.05 in bold

*DFS* disease-free survival, *DSS* disease-specific survival, *HR* hazard ratio, *CI* confidence interval

^a^Hazard ratio for DFS adjusted for presence of ulceration, extranodal growth pattern, and ratio of involved/total nodes (L/N) ratio

^b^Hazard ratio for DSS adjusted for sex and L/N ratio

The estimated 5-year DSS was 28, 34, and 66 % for groin, axilla, and neck, respectively (Fig. 1b). Variables associated with DSS in univariate analysis were the location of nodal metastasis, extranodal growth pattern, L/N ratio, and the size of the largest nodal metastasis (Table 3). The multivariable model for DSS revealed neck site of metastasis (*p* = 0.02) (Table 4) and a lower L/N ratio (*p* < 0.001) to be significantly associated with better survival.

## Discussion

Analysis of 149 melanoma patients undergoing curative TLND showed the 5-year DSS to be 37 % for the entire group, which is similar to percentages reported in the literature.^5^,^21^ Univariate and multivariable analysis revealed differences in prognosis for metastasis in the groin, axilla, or neck. Specifically, nodal metastasis located in the neck was associated with significantly better DSS. No statistically significant difference was found for frequency of distant metastases as the first site of recurrence: groin group 78 %, axilla group 76 %, and neck group 55 % (*p* = 0.26).

In the present study, significant prognostic factors for survival in univariate analysis were site of nodal metastasis, extranodal growth pattern, L/N ratio, and size of the largest nodal metastasis. Besides neck site of nodal metastasis, low L/N ratio was found to be an independent predictor for better DSS, a finding that is in agreement with the recent literature.^18^–^20^ Primary melanoma characteristics were not associated with survival, which is consistent with the study of 441 stage IIIB–C melanoma patients by Balch et al.^3^,^5^ Finding longer survival for neck site metastasis seems contrary to the observation that head and neck melanomas have a worse prognosis than melanomas at other sites.^12^,^13^ However, the literature currently lacks specific studies regarding the prognostic value of the site of nodal metastasis. Moreover, a recent study on the outcome of TLND in stage III melanoma patients with an unknown primary melanoma did notice a survival benefit for patients with a neck metastasis compared to groin or axillary metastasis.^21^

The better prognosis for patients with neck metastasis could be explained by earlier detection of nodal metastasis, resulting in a smaller tumor burden at time of the TLND, and of recurrent locoregional disease in the neck, because of the more superficial and notable position of nodes compared to those in the groin or axilla. In support of this, we found that the lymph node metastases in the neck group were significantly smaller than in the groin and axilla groups. In addition, there was a tendency for patients in the neck group to present more frequently with local or locoregional recurrence as the first sign of progressive disease, rather than distant disease, compared to the groin and axilla groups. However, with the current study size, this tendency did not reach statistical significance.

To evaluate the outcomes of nodal metastasis at different locations without the detection benefit of superficial macrometastasis, we performed a subanalysis of data of 117 patients who underwent completion lymph node dissection shortly after positive sentinel lymph node biopsy in a study by de Vries et al.^22^ This subanalysis showed a 5-year DSS of 63, 68, and 75 % for the groin, axilla, and neck groups, respectively. Although the difference in survival was not statistically significant, the more favorable number for metastasis at the neck site is notable. Therefore, we concluded that the detection benefit alone, even though it proved to be important, could not fully explain the survival difference. Another hypothesis that could explain our findings is the effect of a more extensive lymphatic system in the neck region, which could keep metastases from hematogenous spread. In this case, we would expect differences in the percentage of patients whose disease was upstaged with PET or CT after presenting with palpable lymph node metastases at the different locations. However, in a previous study, we found no differences in the percentage of upstaging between the groups of patients with groin, axilla, or neck metastases (18.3 % groin, 31.3 % axilla, and 23.3 % neck; *p* = 0.12).^14^ The exact mechanisms underlying better survival thus remain unknown. However, possibilities include differences in the behavior of the primary melanoma, a lower detection threshold, immunological advantages of the nodal basin in the neck, and dissection effects.

The findings of this study are limited by the rather small group of patients who underwent TLND of the neck (*n* = 22). Therefore, definitive establishment of the more favorable prognosis for macrometastasis when located in the neck needs confirmation by larger series.

In conclusion, this study showed better prognosis after TLND for stage IIIB–C melanoma when the lymph node metastasis is located in the neck compared to axillary and groin sites.

## References

[1] Dutch Comprehensive Cancer Centers. 2011. http://ikcnet.nl/. Accessed August 2011.

[2] van der Aa MA, de Vries E, Hoekstra HJ, Coebergh JW, Siesling S (2011). Sociodemographic factors and incidence of melanoma in the Netherlands, 1994–2005. Eur J Cancer..

[3] Balch CM, Gershenwald JE, Soong SJ, Thompson JF, Atkins MB, Byrd DR (2009). Final version of 2009 AJCC melanoma staging and classification. J Clin Oncol..

[4] Francken AB, Bastiaannet E, Hoekstra HJ (2005). Follow-up in patients with localised primary cutaneous melanoma. Lancet Oncol..

[5] Balch CM, Gershenwald JE, Soong SJ, Thompson JF, Ding S, Byrd DR (2010). Multivariate analysis of prognostic factors among 2,313 patients with stage III melanoma: comparison of nodal micrometastases versus macrometastases. J Clin Oncol..

[6] Balch CM, Soong SJ, Bartolucci AA, Urist MM, Karakousis CP, Smith TJ (1996). Efficacy of an elective regional lymph node dissection of 1 to 4 mm thick melanomas for patients 60 years of age and younger. Ann Surg..

[7] Cascinelli N, Morabito A, Santinami M, MacKie RM, Belli F (1998). Immediate or delayed dissection of regional nodes in patients with melanoma of the trunk: a randomised trial. WHO Melanoma Programme. Lancet..

[8] Hughes TM, A’Hern RP, Thomas JM (2000). Prognosis and surgical management of patients with palpable inguinal lymph node metastases from melanoma. Br J Surg..

[9] van Akkooi AC, Bouwhuis MG, van Geel AN, Hoedemaker R, Verhoef C, Grunhagen DJ (2007). Morbidity and prognosis after therapeutic lymph node dissections for malignant melanoma. Eur J Surg Oncol..

[10] Balch CM, Soong SJ, Gershenwald JE, Thompson JF, Reintgen DS, Cascinelli N (2001). Prognostic factors analysis of 17,600 melanoma patients: validation of the American Joint Committee on cancer melanoma staging system. J Clin Oncol..

[11] Kruijff S, Bastiaannet E, Kobold AC, van Ginkel RJ, Suurmeijer AJ, Hoekstra HJ (2009). S-100B concentrations predict disease-free survival in stage III melanoma patients. Ann Surg Oncol..

[12] Murali R, Desilva C, Thompson JF, Scolyer RA (2011). Factors predicting recurrence and survival in sentinel lymph node–positive melanoma patients. Ann Surg..

[13] Lachiewicz AM, Berwick M, Wiggins CL, Thomas NE (2008). Survival differences between patients with scalp or neck melanoma and those with melanoma of other sites in the surveillance, epidemiology, and end results (SEER) program. Arch Dermatol..

[14] Bastiaannet E, Wobbes T, Hoekstra OS, van der Jagt EJ, Brouwers AH, Koelemij R (2009). Prospective comparison of [^18^F] fluorodeoxyglucose positron emission tomography and computed tomography in patients with melanoma with palpable lymph node metastases: diagnostic accuracy and impact on treatment. J Clin Oncol..

[15] Poos HP, Kruijff S, Bastiaannet E, van Ginkel RJ, Hoekstra HJ (2009). Therapeutic groin dissection for melanoma: risk factors for short term morbidity. Eur J Surg Oncol..

[16] Bastiaannet E, Beukema JC, Hoekstra HJ (2005). Radiation therapy following lymph node dissection in melanoma patients: treatment, outcome and complications. Cancer Treat Rev..

[17] Henderson MA, Burmeister B, Thompson JF, Di Iulio J, Fisher R, Hong A, et al. Adjuvant radiotherapy and regional lymph node field control in melanoma patients after lymphadenectomy: results of an intergroup randomized trial (ANZMTG 01.02/TROG 02.01). *Ann Surg Oncol.* 2009;27(18S):LBA9084.

[18] Rossi CR, Mocellin S, Pasquali S, Pilati P, Nitti D (2008). N-ratio: a novel independent prognostic factor for patients with stage-III cutaneous melanoma. Ann Surg Oncol..

[19] Xing Y, Badgwell BD, Ross MI, Gershenwald JE, Lee JE, Mansfield PF (2009). Lymph node ratio predicts disease-specific survival in melanoma patients. Cancer..

[20] Berger AC, Fierro M, Kairys JC, Berd D, Sato T, Andrel J (2012). Lymph node ratio is an important and independent prognostic factor for patients with stage III melanoma. J Surg Oncol..

[21] Prens SP, van der Ploeg AP, van Akkooi AC, van Montfort CA, van Geel AN, de Wilt JH (2011). Outcome after therapeutic lymph node dissection in patients with unknown primary melanoma site. Ann Surg Oncol..

[22] de Vries M, Speijers MJ, Bastiaannet E, Plukker JT, Brouwers AH, van Ginkel RJ (2011). Long-term follow-up reveals that ulceration and sentinel lymph node status are the strongest predictors for survival in patients with primary cutaneous melanoma. Eur J Surg Oncol..

